# Supplementary material for: Microbiota Analysis and Characterisation of the Novel Limosilactobacillus Strains Isolated from Dogs
Source: Microorganisms. 2025 May 1;13(5):1059. doi: 10.3390/microorganisms13051059 (PMC12114587; doi:10.3390/microorganisms13051059)
Supplement: Supplementary file 1 [file microorganisms-13-01059-s001.zip › microorganisms-3583189-supplementary.pdf]

Table S1. Signalment information of study population

Dog number	Breed	Age (month) [Baby (<2)/Adult (>12)]	Symptom	Weight (Kg) [Small (<2 Kg)/medium (2–7 Kg)/large (>7 Kg)]
1	Mixed-breed	A	NS	M
2	Mixed-breed	A	NS	L
3	Mixed-breed	A	NS	M
4	Mixed-breed	A	NS	L
5	Mixed-breed	A	NS	M
6	Mixed-breed	A	NS	M
7	Mixed-breed	A	NS	M
8	Mixed-breed	A	NS	M
9	Mixed-breed	A	NS	M
10	Mixed-breed	A	NS	M
11	Mixed-breed	A	NS	M
12	Mixed-breed	A	NS	L
13	Mixed-breed	A	NS	L
14	Mixed-breed	A	NS	L
15	Mixed-breed	A	NS	L
16	Mixed-breed	A	NS	L
17	Mixed-breed	A	NS	L
18	Mixed-breed	A	HT	M
19	Mixed-breed	A	NS	L
20	Mixed-breed	A	NS	L
21	Mixed-breed	A	NS	L
22	Mixed-breed	A	NS	L
23	Mixed-breed	A	NS	L
24	Mixed-breed	A	NS	M
25	Mixed-breed	A	NS	M
26	Mixed-breed	A	NS	M
27	Mixed-breed	A	NS	M
28	Mixed-breed	A	NS	M
29	Mixed-breed	A	NS	M
30	Mixed-breed	A	NS	M
31	Mixed-breed	B	NS	S
32	Mixed-breed	B	NS	S
33	Mixed-breed	B	NS	S
34	Mixed-breed	B	NS	S
35	Mixed-breed	B	NS	S
36	Mixed-breed	B	NS	S
37	Mixed-breed	B	NS	S
38	Mixed-breed	B	NS	S
39	Mixed-breed	B	NS	S
40	Mixed-breed	B	NS	S

A, adult; B, baby; NS, no symptoms; HT, hematochezia, S, small; M, medium; L, large.

Table S2. Quality of DNA extracted from dog feces

Name	260 Raw	280 Raw	320 Raw	260	280	260/280	ng/μL
B3	0.085	0.066	0.051	0.025	0.013	1.9	24.7
B5	0.084	0.068	0.059	0.016	0.008	2.05	15.863
B7	0.079	0.06	0.046	0.024	0.012	1.999	23.535
A2	0.084	0.06	0.042	0.033	0.016	2.03	33.076
A4	0.104	0.071	0.044	0.05	0.024	2.06	49.728
A10	0.128	0.105	0.08	0.038	0.023	1.972	38.104

Feces from puppy dog B3, B5 and B7; adult dog A2, A4 and A10.

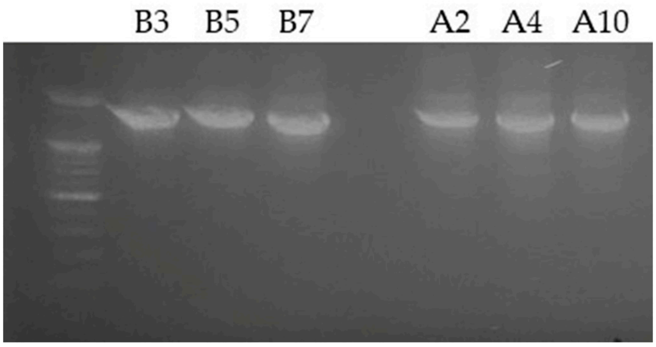

Figure S1. Electrophoresis results from DNA extracted from dog feces (feces from puppy dog B3, B5, and B7; adult dog A2, A4, and A10).

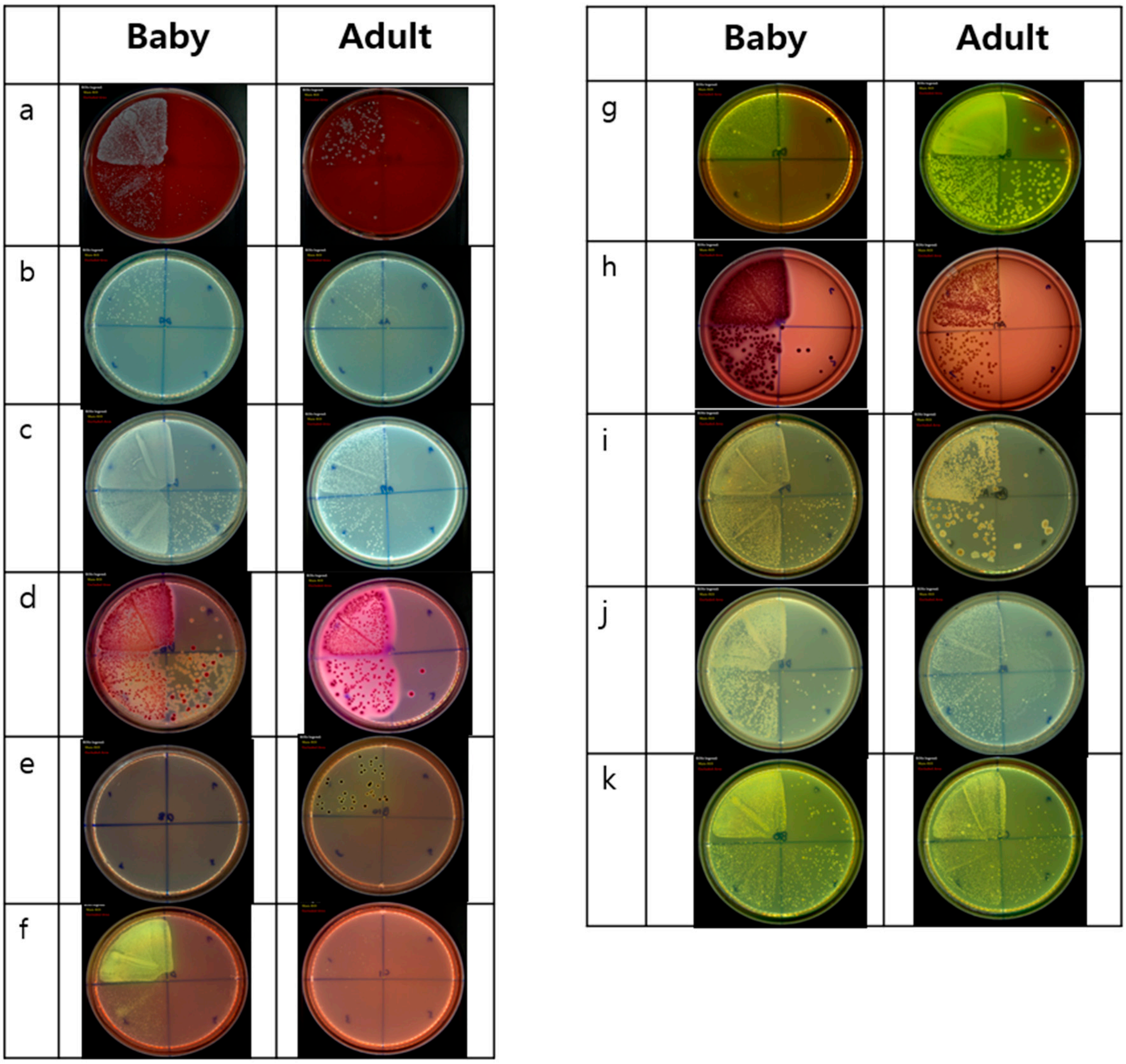

Figure S2. Diversity and strain-specific characteristics of gut microbiota, as revealed by culturing faecal samples on various media. Bacterial colony counts on a) Blood agar, b) TSN, c) PDA, d) MC, e) SS, f) MS, g) BG, h) MACS, i) MRS, j) TOS, and k) PCA with BCP media of adult dogs and puppies. Abbreviations: TSN, tryptone sulfite neomycin; PDA, potato dextrose agar; MC, MacConkey; SS, salmonella-shigella; MS, mannitol salt; BG, brilliant green; MACS, MacConkey sorbitol; MRS, De Man, Rogosa, and Sharpe; TOS, transgalactosylated oligosaccharides with MUP; PCA, plate count agar; BCP, bromocresol purple.
