# Supplementary material for: Microbiota Analysis and Characterisation of the Novel Limosilactobacillus Strains Isolated from Dogs
Source: Microorganisms. 2025 May 1;13(5):1059. doi: 10.3390/microorganisms13051059 (PMC12114587; doi:10.3390/microorganisms13051059)
Supplement: Supplementary file 1 [file microorganisms-13-01059-s001.zip › Supplementary Figure S3_L. reuteri JJ37.pdf]

File: 37_785F.ab1 Run Ended: 2023/6/30 21:36:29 Signal G:1776 A:2124 C:3162 T:2236

Sample: 37_785F Lane: 11 Base spacing: 15.212056 1493 bases in 18479 scans

10 20 30 40 50 60 70 80 90 100 110 120

T TAA AATG AGTGCTA CGTGTT GGAGGGTTTCCGCCCTTCAGTGCCGGAGCTAACGCATTAAGCACTCCGCCTGGGG AGT ACGACCGCAAGGTTGAAACTCAAAGGAATT GACGGGGGCCCGCA

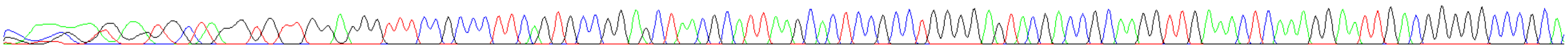

130 140 150 160 170 180 190 200 210 220 230 240

CAAGCGGTGGAGCATGTGGTTTAATTCGAAGCTACGCGAAGAACCTTACCAGGTCTTGACATCTTGCGCTAACCTTAGAGATAAGGCGTTCCCTTCGGGGACGCAATGACAGGTGGTGCATGGT

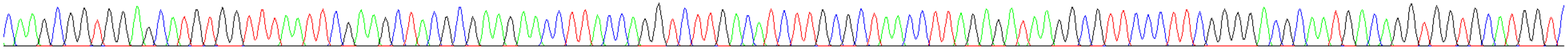

250 260 270 280 290 300 310 320 330 340 350 360 370

CGTCGTCAGCTCGTGTCGTGAGATGTTGGGTTAAGTCCCGCAACGAGCGCAACCCTTGTTACTAGTTGCCAGCATTAAGTTGGGCACTCTAGTGAGACTGCCGGTGACAAACCGGAGGAAGGTGG

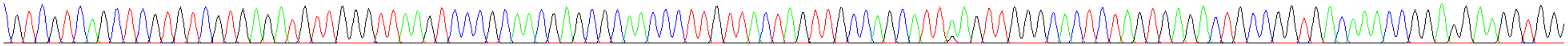

380 390 400 410 420 430 440 450 460 470 480 490

GGACGACGTCAGATCATCATGCCCCTTATGACCTGGGCTACACACGTGCTACAATGGACGGTACAACGAGTCGCAAACTCGCGAGAGTAAGCTAATCTCTTAAAGCCGTTCTCAGTTCGGACTGT

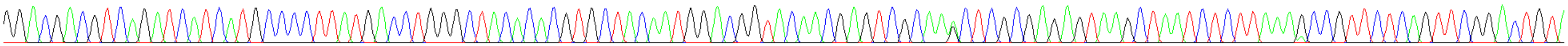

500 510 520 530 540 550 560 570 580 590 600 610 620

AGGCTGCAACTCGCCTACACGAAGTCGGAATCGCTAGTAATCGCGGATCAGCATGCCGCGGTGAATACGTTCCCGGGCCTTGTACACACCGCCCGTCACACCATGGGAGTTTGTAACGCCCAAA

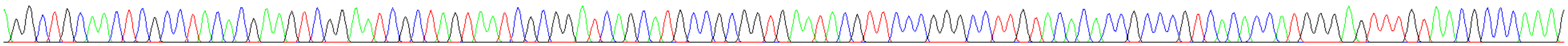

630 640 650 660 670 680 690 700 710 720 730 740

GTCGGTGGCCTAACCTTTATGGAGGGAGCCGCCTAAGGCGGGACAGATGACTGGGGTGAAGTCGTAACAAGGTAGCCGTAGGAGAACCTGCGGCTGGATCACCCCCCCTTTTTATAAAAAGGCC A

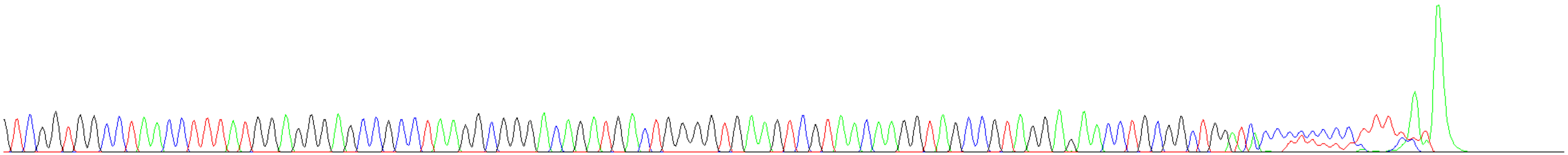

750 760 770 780 790 800 810 820 830 840 850 860 870

AAGTGGTATCCT CGTTCT CCATTG TTAACGAAGGCCGTAAA GAT GTGTTAC CCAACAGGGTAATCAATCACCCGGTAATCGAATGTCGACTACAACACACAGGTCTT CGGCT GGCACTCGTTCTC

880 890 900 910 920 930 940 950 960 970 980 990

CCTGTTAGCCCTGCGCTCC GCCTGTATTCCGTCTGACTCGGAGGAA GCTTCCAC CCTGTTACTACACTCAAAATGTGTTCGGCGGG G GGCCCACAAAAAAC AGCGAAATTGCGTCTTATTAGT

1000 1010 1020 1030 1040 1050 1060 1070 1080 1090 1100 1110 1120

TTATTATGTCTCCGCCGCAAGGCCTA GCGTGAT CACCACCTCTCTTCTAGATAATAAACAATAGGTGCCCTGCC CCCGAGGCCCAAAGTGCAGTTGTGT CCTTTGCACTTCACTTCATTGAGAAT

1130 1140 1150 1160 1170 1180 1190 1200 1210 1220 1230 1240

CAGATTTTTTTTTCTTTAAGTCCCCGC CTCCCGCCCCCCCCCCTTGCC CCCATGAGTCAAACAT CCGTTGGGCCCTCCCCAATGAAACAGGAG GACACAAAGGGGGGGGAGGGG GGGGAACA
