# Supplementary material for: Microbiota Analysis and Characterisation of the Novel Limosilactobacillus Strains Isolated from Dogs
Source: Microorganisms. 2025 May 1;13(5):1059. doi: 10.3390/microorganisms13051059 (PMC12114587; doi:10.3390/microorganisms13051059)

File: 68_785F.ab1   Run Ended: 2023/6/30 21:36:29   Signal G:1313 A:1711 C:2725 T:1903

Sample: 68_785F   Lane: 7   Base spacing: 15.316603   1499 bases in 17693 scans

10 20 30 40 50 60 70 80 90 100 110 120

C G CAGATG AG TGCTAGGTGTTGGAGGGTTTCCGCCCTTCAGTGCCGGAGCTAACGCATTAAGCACTCCGCCTGGGGAGTACGACCGCAAGGTTGAAACTCAAAGGAATTGACGGGGGCCCGCACA

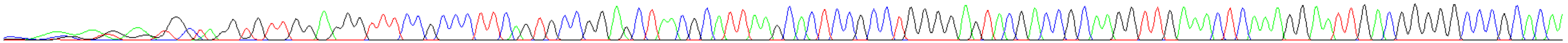

130 140 150 160 170 180 190 200 210 220 230 240 250

AGCGGTGGAGCATGTGGTTTAATTCGAAGCTACGCGAAGAACCTTACCAGGTCTTGACATCTTGCGCTAACCTTAGAGATAAGGCGTTCCCTTCGGGGACGCAATGACAGGTGGTGCATGGTCGT

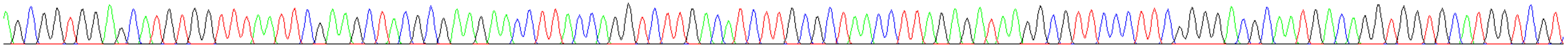

260 270 280 290 300 310 320 330 340 350 360 370

CGTCAGCTCGTGTCGTGAGATGTTGGGTTAAGTCCCGCAACGAGCGCAACCCTTGTTACTAGTTGCCAGCATTAAGTTGGGCACTCTAGTGAGACTGCCGGTGACAAACCGGAGGAAGGTGGGGA

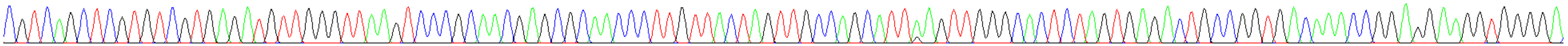

380 390 400 410 420 430 440 450 460 470 480 490 500

CGACGTCAGATCATCATGCCCCTTATGACCTGGGCTACACACGTGCTACAATGGACGGTACAACGAGTCGCAAACTCGCGAGAGTAAGCTAATCTCTTAAAGCCGTTCTCAGTTCGGACTGTAGG

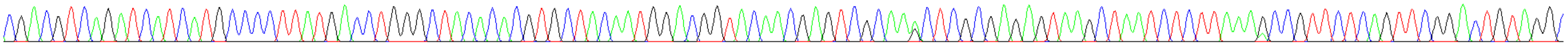

510 520 530 540 550 560 570 580 590 600 610 620

CTGCAACTCGCCTACACGAAGTCGGAATCGCTAGTAATCGCGGATCAGCATGCCGCGGTGAATACGTTCCCGGGCCTTGTACACACCGCCCGTCACACCATGGGAGTTTGTAACGCCCAAAGTCG

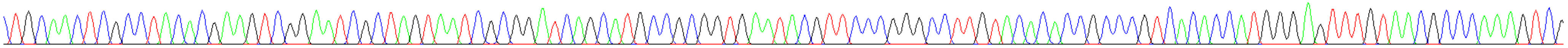

File: 68_785F.ab1 Run Ended: 2023/6/30 21:36:29 Signal G:1313 A:1711 C:2725 T:1903

Sample: 68_785F Lane: 7 Base spacing: 15.316603 1499 bases in 17693 scans

630 640 650 660 670 680 690 700 710 720 730 740

GTGGCCTAACCTTTATGGAGGGAGCCGCCTAAGGCGGGACAGATGACTGGGGTGAAGTCGTAACAAGGTAGCCGTAGGAGAACCTGCGGCTGGATCCCCC C CCTTTT AAAA AGCGGTAAAAAG

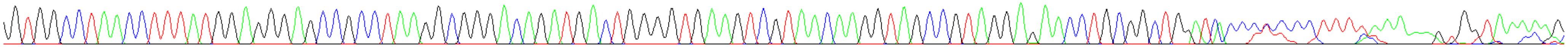

750 760 770 780 790 800 810 820 830 840 850 860 870

CTGTTTTCCTGGACTTAAACCTGACTTTTAAGGCATGAAAACCCAGGGGTTTGCAAA CGGGTTT AGTACTCTGGGGAGGCACGCCCCGAACAACAATGAGCACACCTTGGGTGGGTTTTCCCC CC

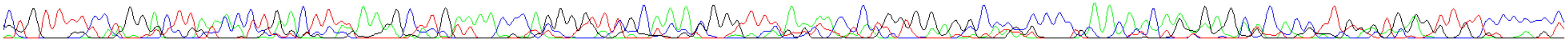

880 890 900 910 920 930 940 950 960 970 980 990

ATCAGCCCGGGACTGAAACAGTAACAACACC CACGCTGGGGGAGTTACCA ACCGAGTGTATGACC C ACGAGGTTACTGGGGGTGCCCCCCAAAAAGGGG GAGA A GGGTTTTTTTTATTAT

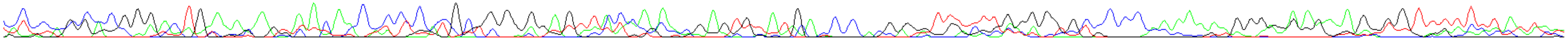

1000 1010 1020 1030 1040 1050 1060 1070 1080 1090 1100 1110

ATATCT ACAAAAAATTACCAGGACTTTTTTTTTT TTT CTTTTTCTTGATGAAA AACAGTT CCTGTTTTTCAAGACGAAAAAC AAAGGAGGTGTGTGACCACGAACCTTCCTCATCCAAAGACA

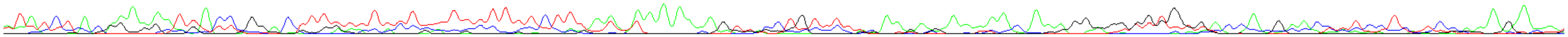

1120 1130 1140 1150 1160 1170 1180 1190 1200 1210 1220 1230 1240

TG T TTT TTTT CGCCTACC AACCGGCAACCC CCCTCCCCCCTTGTC ACC AAATAAAAAAAATATTTCCTTCCGCCATCCCCCGGAGAAAAACGCGGCAAC AAAC CTGGGGGGAA GGGGGGG AAAAAAAT

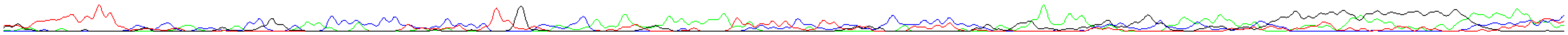

Supplement: Supplementary file 1 [file microorganisms-13-01059-s001.zip › Supplementary Figure S4_L. reuteri JJ68.pdf]
