# Supplementary material for: Microbiota Analysis and Characterisation of the Novel Limosilactobacillus Strains Isolated from Dogs
Source: Microorganisms. 2025 May 1;13(5):1059. doi: 10.3390/microorganisms13051059 (PMC12114587; doi:10.3390/microorganisms13051059)
Supplement: Supplementary file 1 [file microorganisms-13-01059-s001.zip › Supplementary Figure S5_L. reuteri JJ69.pdf]

File: 69_907R.ab1 Run Ended: 2023/6/30 21:36:29 Signal G:1468 A:1787 C:3845 T:2620

Sample: 69_907R Lane: 1 Base spacing: 15.617066 1377 bases in 16416 scans

CAGTTCGGCGTCTCCCAGGCGGAGTGCTTAATGCGTTAGCTCCGGCACTGAAGGGCGGAAACCCTCCAACACCTAGCACTCATCGTTTACGGCATGGACTACCAGGGTATCTAATCCTGTTCGCTAC

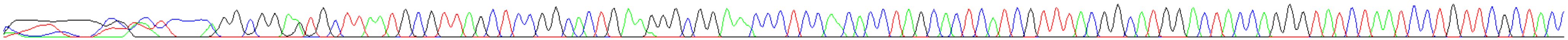

CCATGCTTTCGAGCCTCAGCGTCAGTTGCAGACCAGACAGCCGCCTTCGCCACTGGTGTTCTTCCATATATCTACGCATTCCACCGCTACACATGGAGTTCCACTGTCCTCTTCTGCACTCAAGTC

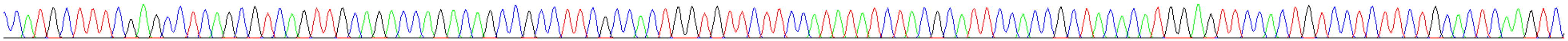

GCCCGGTTTCCGATGCACTTCTTCGGTTAAGCCGAAGGCTTTCACATCAGACCTAAGCAACCGCCTGCGCTCGCTTTACGCCCAATAAATCCGGATAACGCTTGCCACCTACGTATTACCGCGGCT

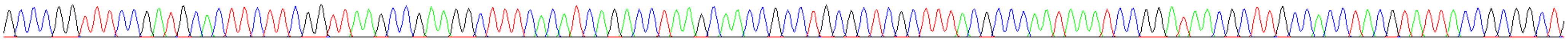

GCTGGCACGTAGTTAGCCGTGACTTTCTGGTTGGATACCGTCACTGCGTGAACAGTTACTCTCACGCACGTTCTTCTCCAACAACAGAGCTTTACGAGCCGAAACCCTTCTTCACTCACGCGGTGT

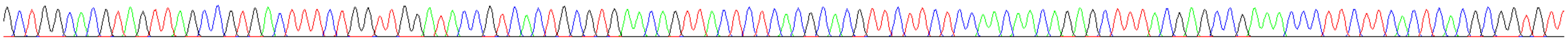

TGCTCCATCAGGCTTGCGCCCATTGTGGAAGATTCCCTACTGCTGCCTCCCGTAGGAGTATGGACCGTGTCTCAGTTCCATTGTGGCCGATCAGTCTCTCAACTCGGCTATGCATCATCGCCTTGG

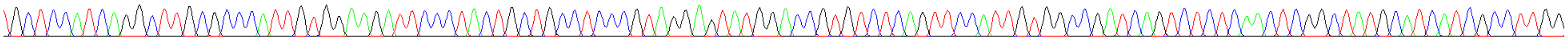

File: 69_907R.ab1 *Run Ended: 2023/6/30 21:36:29* *Signal G:1468 A:1787 C:3845 T:2620*

Sample: 69_907R *Lane: 1* *Base spacing: 15.617066* *1377 bases in 16416 scans*

640 650 660 670 680 690 700 710 720 730 740 750

TAAGCCGTTACCTTACCAACTAGCTAATGCACCGCAGGTCCATCCCAGAGTGATAGCCAAAGCCATCTTTCAAACAAAAGCCATGTGGCTTTTGTTGTTATGCGGTATTAGCATCTGTTTCCAA

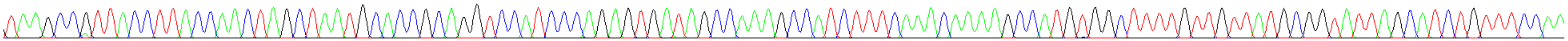

760 770 780 790 800 810 820 830 840 850 860 870

ATGTTATCCCCCGCTCCGGGGCAGGTTACCTACGTGTTACTCACCCGTCCGCCACTCACTGGTGATCCATCGTCAATCAGGTGCAAGCACCATCAATCAGTTGGGCCAGTGCGTACGACTTGCA

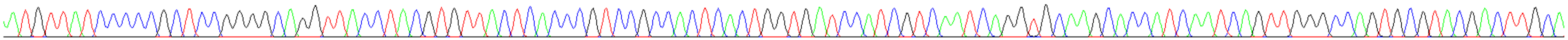

880 890 900 910 920 930 940 950 960 970 980 990 1000

TGTATTAGGCACACCGCCGGCGTTCATCCTGAGCCATGATCAACTCTATGCGCCGCTTTTTTAACCAAAATACGCCCACTAACGCGCGTATAGTGAAGAAAACCATCACTAAACAACCTGTA

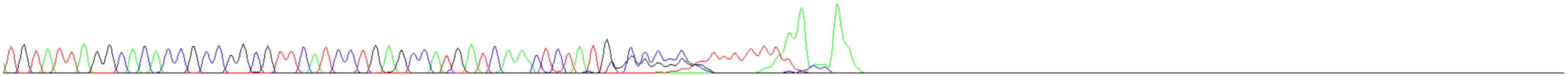

1010 1020 1030 1040 1050 1060 1070 1080 1090 1100 1110 1120

AGGCCGGGAGGGCAGCTAACAGGAAAGTCAGGGCAATCGTGGCGGGGGCCGGGAAAAAGTAGGGGGCGAGCAGTACACGGTAATTTTGGGGGAACAGGGGACGTCGGTGGGTACCTAAGT

1130 1140 1150 1160 1170 1180 1190 1200 1210 1220 1230 1240 1250

GCGAATGTAGAAAGCCTTTCGGGCTAAGGGAAAAAAATATCGGAGACGGGGCGCCACGCTGGGGGCAGGGGGCACGCGAAGTCCTCAGGTTGCGGGAGGATGAATCGCCATCTTTCCGGAGATTATATCACAGCGTG
