# Supplementary material for: Microbiota Analysis and Characterisation of the Novel Limosilactobacillus Strains Isolated from Dogs
Source: Microorganisms. 2025 May 1;13(5):1059. doi: 10.3390/microorganisms13051059 (PMC12114587; doi:10.3390/microorganisms13051059)
Supplement: Supplementary file 1 [file microorganisms-13-01059-s001.zip › Supplementary Figure S6_L. reuteri JJ71.pdf]

File: 71_907R.ab1 Run Ended: 2023/6/30 21:36:29 Signal G:1038 A:1242 C:2709 T:1889

Sample: 71_907R Lane: 14 Base spacing: 15.274146 1402 bases in 18455 scans

10 20 30 40 50 60 70 80 90 100 110 120

C GGTCGG CGTCTCCCAGGCGGAGTGCTTAATGCGTTAGCTCCGGCACTGAAGGGCGGAAACCCTCCAACACCTAGCACTCATCGTTTACGGCATGGACTACCAGGGTATCTAATCCTGTTCGCTACC

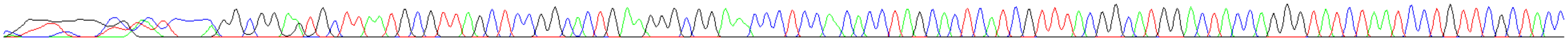

130 140 150 160 170 180 190 200 210 220 230 240 250

CATGCTTTCGAGCCTCAGCGTCAGTTGCAGACCAGACAGCCGCCTTCGCCACTGGTGTTCTTCCATATATCTACGCATTCCACCGCTACACATGGAGTTCCACTGTCCTCTTCTGCACTCAAGTCG

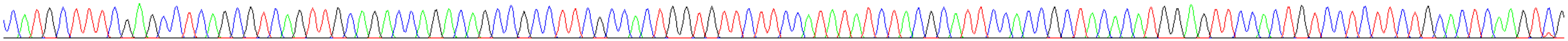

260 270 280 290 300 310 320 330 340 350 360 370

CCCGGTTTCCGATGCACTTCTTCGGTTAAGCCGAAGGCTTTCACATCAGACCTAAGCAACCGCCTGCGCTCGCTTTACGCCCAATAAATCCGGATAACGCTTGCCACCTACGTATTACCGCGGCTG

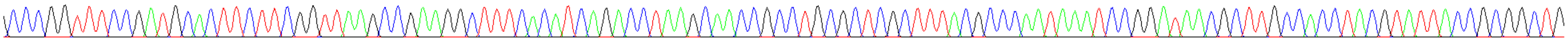

380 390 400 410 420 430 440 450 460 470 480 490 500

CTGGCACGTAGTTAGCCGTGACTTTCTGGTTGGATACCGTCACTGCGTGAACAGTTACTCTCACGCACGTTCTTCTCCAACAACAGAGCTTTACGAGCCGAAACCCTTCTTCACTCACGCGGTGTT

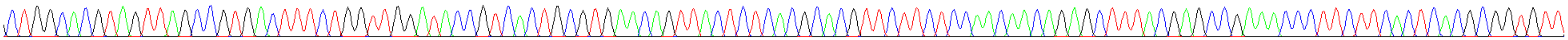

510 520 530 540 550 560 570 580 590 600 610 620 630

GCTCCATCAGGCTTGCGCCCATTGTGGAAGATTCCCTACTGCTGCCTCCCGTAGGAGTATGGACCGTGTCTCAGTTCCATTGTGGCCGATCAGTCTCTCAACTCGGCTATGCATCATCGCCTTGGT

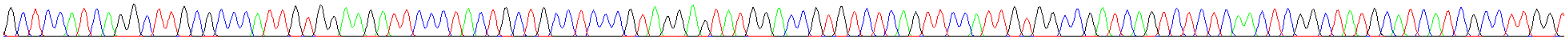

File: 71_907R.ab1 *Run Ended: 2023/6/30 21:36:29* *Signal G:1038 A:1242 C:2709 T:1889*

Sample: 71_907R *Lane: 14* *Base spacing: 15.274146* *1402 bases in 18455 scans*

640 650 660 670 680 690 700 710 720 730 740 750

AAGCCGTTACCTTACCAACTAGCTAATGCACCGCAGGTCCATCCCAGAGTGATAGCCAAAGCCATCTTTCAAACAAAAGCCATGTGGCTTTTGTTGTTATGCGGTATTAGCATCTGTTTCCAAA

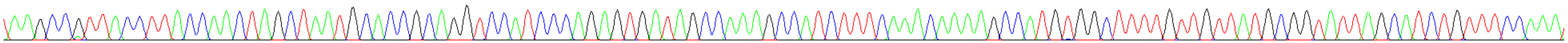

760 770 780 790 800 810 820 830 840 850 860 870 880

TGTTATCCCCCGCTCCGGGGCAGGTTACCTACGTGTTACTCACCCGTCCGCCACTCACTGGTGATCCATCGTCAATCAGGTGCAAGCACCATCAATCAGTTGGGCCAGTGCGTACGACTTGCATG

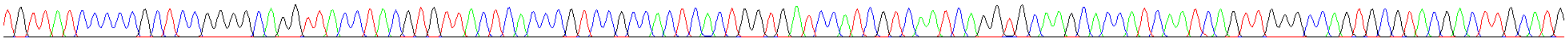

890 900 910 920 930 940 950 960 970 980 990

TATTAGGCACACCGCCGGCGTTCATCCTGAGCCATGATCAACTCTATGCCGCCCTGTTTTAAAAAAATTCCCCTCCAAAGGCGGGTGTTGGTGATACAGTTGTCAAGAAATGTTCCG

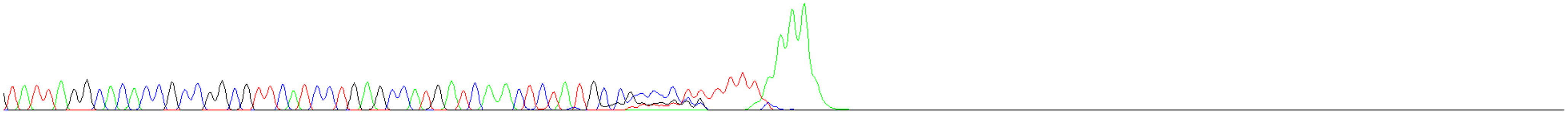

1000 1010 1020 1030 1040 1050 1060 1070 1080 1090

GTGGGCGGAAGCTACAAAAAAAAGGAAAAGACCTGCTGCCGCCCGGATAAGGGGGGGGAGGGTCAGGATATTTCGAGATGATAATGTGGGGTTAATCGCAA

1100 1110 1120 1130 1140 1150 1160 1170 1180 1190

ACTAAGCCTTGTTAGGTAAAAGTACAACCGGCCGCTGGAGAAAAGGGAAGGGGGGGAGGGGGAGAAAACCATCCTTTGCTAACCCTTGGGGA
