# Supplementary material for: Microbiota Analysis and Characterisation of the Novel Limosilactobacillus Strains Isolated from Dogs
Source: Microorganisms. 2025 May 1;13(5):1059. doi: 10.3390/microorganisms13051059 (PMC12114587; doi:10.3390/microorganisms13051059)
Supplement: Supplementary file 1 [file microorganisms-13-01059-s001.zip › Supplementary Figure S7_L. reuteri JJ77.pdf]

File: 77_785F.ab1 Run Ended: 2023/6/30 21:36:29 Signal G:1778 A:1836 C:2704 T:1838

Sample: 77_785F Lane: 12 Base spacing: 15.153604 1619 bases in 19214 scans

10 20 30 40 50 60 70 80 90 100 110 120

CTAAGGATGAGTGCTACGTGTTGGAGGGTTTCCGCCCTTCAGTGCCGGAGCTAACGCATTAAGCACTCCGCCTGGGGAGTACGACCGCAAGGTTGAAACTCAAAGGAATTGACGGGGGCCCGCAC

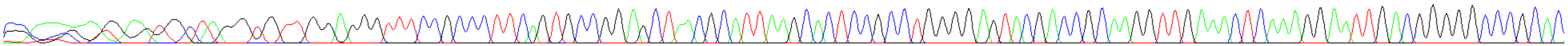

130 140 150 160 170 180 190 200 210 220 230 240 250

AAGCGGTGGAGCATGTGGTTTAATTCGAAGCTACGCGAAGAACCTTACCAGGTCTTGACATCTTGCGCTAACCTTAGAGATAAGGCGTTCCCTTCGGGGACGCAATGACAGGTGGTGCATGGTCG

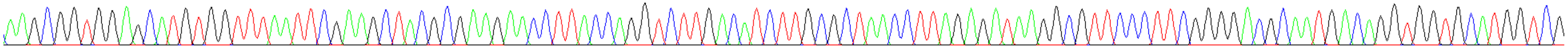

260 270 280 290 300 310 320 330 340 350 360 370

TCGTCAGCTCGTGTCGTGAGATGTTGGGTTAAGTCCCGCAACGAGCGCAACCCTTGTTACTAGTTGCCAGCATTAAGTTGGGCACTCTAGTGAGACTGCCGGTGACAAACCGGAGGAAGGTGGGG

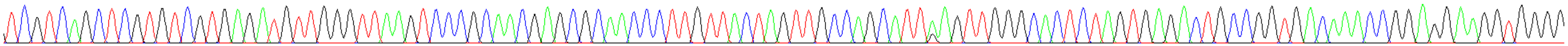

380 390 400 410 420 430 440 450 460 470 480 490 500

ACGACGTCAGATCATCATGCCCCTTATGACCTGGGCTACACACGTGCTACAATGGACGGTACAACGAGTCGCAAGCTCGCGAGAGTAAGCTAATCTCTTAAAGCCGTTCTCAGTTCGGACTGTAG

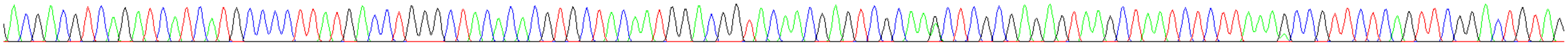

510 520 530 540 550 560 570 580 590 600 610 620

GCTGCAACTCGCCTACACGAAGTCGGAATCGCTAGTAATCGCGGATCAGCATGCCGCGGTGAATACGTTCCCGGGCCTTGTACACACCGCCCGTCACACCATGGGAGTTTGTAACGCCCAAAGT

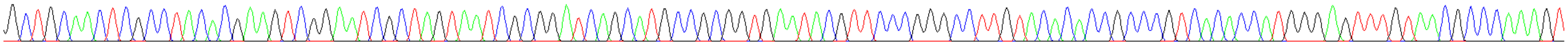

File: 77_785F.ab1 Run Ended: 2023/6/30 21:36:29 Signal G:1778 A:1836 C:2704 T:1838

Sample: 77_785F Lane: 12 Base spacing: 15.153604 1619 bases in 19214 scans

630 640 650 660 670 680 690 700 710 720 730 740

CGGTGGCCTAACCTTTATGGAGGGAGCCGCCTAAGGCGGGACAGATGACTGGGGTGAAGTCGTAACAAGGTAGCCGTAGGAGAACCTGCGGCTGGATCACCCCCCCCTTTTAAAAA ATT TAAACC

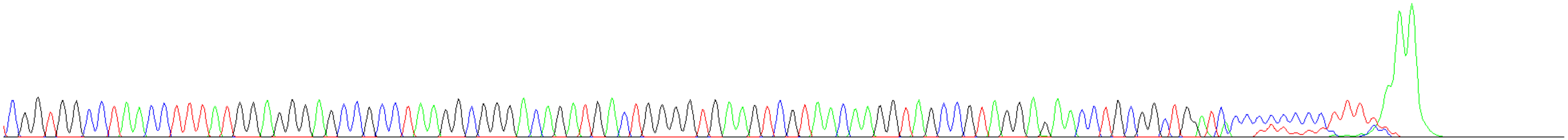

750 760 770 780 790 800 810 820 830 840 850 860 870

T TTTTCCGT ATGGT TTCC CCCGTTCTCGAACAATT AACACCCGCGTAGCAC ACGCGTTC GCCACACTTTGGTCATCCCCCGC CCTACAC ATG CACCTGCAC CCTCCTGTATTGTGCTCGCC

880 890 900 910 920 930 940 950 960 970 980 990

CTAC TCC TC GTGTGTATGAA CGCT ACC GC CGCCTCCATCACAATCTATCAATAATCATATCGACCCA CTCGGCTATTTCAATA CAACT ATCAACAAAGTGAGGGGGCTGGTCTGAC CGCTCAAA

1000 1010 1020 1030 1040 1050 1060 1070 1080 1090 1100 1110 1120

ATCACGAGAGAG GACCCGT CCGCGT TC CTACCC AATGTCG TCGCTCA GACGGAAAAGACCC GGGGCT AATCGAG TTTTAACAT AAGG TAATGCCTT CC CCCAGATTCCTAG ATTAAACCAACGAACTTT

1130 1140 1150 1160 1170 1180 1190 1200 1210 1220 1230 1240

TTTCGTGGCCC CA GATCCTTACAAT AGCC ATTAAGGCTGGGTGA AA AC TA C C ATTTTT TCGC ACGGGGCT CTCTCCAGAGGAAA GGGG GAATAG CGGGCTATA G GGTGGT GA GAAGCAT AATCT
